# Do trial benefits predict real-world gains in metastatic castration resistant prostate cancer

**DOI:** 10.1093/jncics/pkaf018

**Published:** 2025-02-13

**Authors:** Sarah Axeen, Alice J Chen, Darius N Lakdawalla, Neal Masia, Alexander Niyazov, Bhakti Arondekar, Stephen J Freedland

**Affiliations:** Leonard D. Schaeffer Center for Health Policy and Economics, University of Southern California, Los Angeles, CA 90089, United States; Keck School of Medicine, University of Southern California, Los Angeles, CA 90089, United States; Leonard D. Schaeffer Center for Health Policy and Economics, University of Southern California, Los Angeles, CA 90089, United States; Sol Price School of Public Policy, University of Southern California, Los Angeles, CA 90089, United States; Leonard D. Schaeffer Center for Health Policy and Economics, University of Southern California, Los Angeles, CA 90089, United States; Sol Price School of Public Policy, University of Southern California, Los Angeles, CA 90089, United States; Alfred E. Mann School of Pharmacy and Pharmaceutical Sciences, University of Southern California, Los Angeles, CA 90089, United States; Columbia Business School, Columbia University, New York City, NY 10027, United States; Pfizer, Inc., Collegeville, PA 19426, United States; Pfizer, Inc., Collegeville, PA 19426, United States; Department of Urology, Cedars Sinai Medical Center, Los Angeles, CA 90048, United States

## Abstract

**Background:**

It is important to understand the relationship between drug efficacy measured in randomized clinical trials (RCTs) and real-world drug effectiveness. We estimate how RCT overall survival (OS) and RCT radiographic progression-free survival (rPFS) benefits predict the association between treatments and real-world OS gains for metastatic castration-resistant prostate cancer (mCRPC) drugs.

**Methods:**

Using the National Cancer Institute list of approved cancer drugs and the National Comprehensive Cancer Network Treatment Guidelines, we identified all pharmaceutical therapies for mCRPC approved between 2010 and 2019. We obtained RCT OS and rPFS hazard ratios from the pivotal trials used for Food and Drug Administration (FDA) approval, and we estimated real-world OS hazard ratios using the Optum Clinformatics Extended DataMart Databases. We modeled real-world OS hazard ratios as a function of both RCT OS and RCT rPFS hazard ratios using Cox proportional hazards regressions, adjusted for year of diagnosis, age, race, and Elixhauser Comorbidity Index.

**Results:**

When we did not account for nonrandom real-world selection of patients into receiving a newly approved therapy (ie, “treatment selection bias”), real-world OS gains were 15% lower than associated RCT OS and RCT rPFS benefits. However, after accounting for treatment selection bias in real-world settings, real-world OS gains were almost 28% greater than RCT OS and RCT rPFS benefits. Association between treatment and OS gains increased the longer a new therapy was on the market.

**Conclusions:**

After adjusting for treatment selection bias, RCT OS and RCT rPFS estimates serve as useful, or even conservative, predictors of RW OS gains.

## Introduction

Data from both a real-world setting and randomized clinical trials (RCTs) are important for understanding the value of new therapies. RCTs measure efficacy under controlled and rigorous circumstances, whereas real-world data offer information on treatment effectiveness in the routine clinical practice setting. For providers, payers, and health technology assessment agencies, it is crucial to understand the degree to which benefits seen in RCTs correlate with outcomes expected in the real world.^1^ In particular, reimbursements and coverage decisions are often made when only RCT data exist, and shortly after a drug is launched, providers must make treatment decisions based on limited real-world data.

There are many reasons why RCT survival may differ from real-world survival.^2^ Whereas RCTs are conducted in an experimental setting with well-designed patient treatment plans, the real-world setting features variability in treatment timing, patient follow-up, and provider oversight. The set of patients receiving treatment may also be more ethnically, socioeconomically, and clinically diverse, including some ineligible for the original RCTs (eg, older, sicker patients). Over time, providers in the real-world may improve their ability to identify patients who will most benefit from a new therapy and their posttreatment management, leading to even greater benefits.^3-5^ Thus, whereas RCTs provide the least biased estimates for the effect of a drug within the controlled clinical conditions and treated population of the study, real-world outcomes may differ from RCT results.

In this article, we examine the extent to which RCT benefits predict real-world associations between treatment receipt and survival gains. We focus on recently approved therapies for metastatic castration-resistant prostate cancer (mCRPC) and 2 commonly used mCRPC endpoints: overall survival (OS), which measures the length of time patients are alive after the start of treatment, and radiographic progression-free survival (rPFS), which measures the length of time during which a patient’s cancer does not progress or worsen, as determined by imaging tests.

Prior work has focused on the relationship between trial outcomes and real-world patients for a specific drug treating 1 cancer type.^6-8^ Studies synthesizing this relationship across many drugs or cancer types considered only OS as the RCT endpoint of interest.^9^ One study analyzed how both RCT OS and RCT PFS relate to real-world outcomes in the Medicare population, but it did not include prostate cancer as 1 of the tumor types studied.^10^ To the best of our knowledge, this study is the first to use commercial claims data to estimate how both RCT OS and RCT rPFS correlate with improved real-world survival across multiple drugs and drug classes within prostate cancer. We estimate the Cox Survival Regression offset term that measures the difference between RCT efficacy and real-world effectiveness for mCRPC patients.^10^ We also test whether the relationship between real-world OS and RCT outcomes changes the longer a new therapy is on the market.

## Methods

### Data sources

To identify the mCRPC drugs, we reviewed the National Cancer Institute’s list of approved drugs and the National Comprehensive Cancer Network Treatment Guidelines. For each drug, we identified the US Food and Drug Administration (FDA) approval date for mCRPC treatment, and we limited our sample to new molecules with indication-specific approval dates between 2010 and 2019. Next, we reviewed each drug’s most recent FDA label to identify the pivotal trials used for approval. From the FDA labels and each pivotal trial’s published studies, we abstracted information on the inclusion and exclusion criteria, RCT OS estimates (for the drug, its comparator, and the corresponding hazard ratio), and RCT rPFS estimates if available.

We calculated real-world OS using patient-level data from January 2009 to December 2020 of the Optum Clinformatics Extended DataMart Databases (hereafter, “Optum”). These data allow us to observe real-world claims at least 1-year prior to our earliest launch (required for the control group discussed below) and 1-year after our latest launch. The Optum database covers more than 13 million annual lives of UnitedHealth Group members, and it includes dates of insurance enrollment, inpatient and outpatient claims, prescription drug fills, patient demographics, and date of death (month/year). For the subset of patients using labs that share data with Optum, we also examined laboratory testing claims.

### Inclusion and exclusion criteria

We followed the Freedland et al. (2021) criteria to identify mCRPC patients in Optum.^11^ We identified male patients aged 18 and older at the index date with at least 1 inpatient service claim, or 2 outpatient service claims separated by at least 30 days, listing a prostate cancer diagnosis. The index date is the date of the first claim with a metastatic disease diagnosis coincident with or after the first claim with a prostate cancer diagnosis. However, if the first claim with evidence of castration-resistance post-dates the first metastatic disease claim, we use the castration-resistance date as the index. We required patients to be continuously enrolled in insurance for at least 180 days prior to their index dates and at least 180 days after diagnosis or up to the date of death. Table S1 shows the corresponding changes in sample size with each identification criterion, and Table S2 provides detailed documentation of the inclusion and exclusion criteria in constructing the mCRPC sample.

Importantly, we enriched our inclusion criteria to match the criteria listed in the pivotal trials. For example, some RCTs included only chemotherapy-naïve patients, whereas others included only patients with prior docetaxel therapy. These additional criteria identified real-world patients more comparable to those enrolled in the corresponding RCTs.

For each pivotal trial, we identified the Optum patients receiving either the treatment regimen of interest after its FDA approval date (“treated”) or its respective comparator regimen (“control”). We limited the treated group to patients treated within 6 years of the approval date to reduce confounding by the adoption of newer therapies or regimens not considered in our sample of drugs. As discussed later, we test the sensitivity of our results to examining only patients treated within 3 years of the approval date and investigate the possibility of confounding from new therapies arriving in years 4- 6.

We identified “control” patients using 2 approaches. The first approach identified patients receiving the comparator therapy after the new therapy had launched (hereafter, “post-launch controls”). However, because treatment decisions in the real world are nonrandom, patients remaining on the comparator after the new drug launches may differ unobservably from those who received it. To mitigate nonrandom treatment selection, our second approach follows prior literature by identifying patients receiving the comparator regimen during the 2 years prior to the new therapy’s launch (hereafter, “pre-launch controls”).^10^ Pre-launch controls include patients who would be eligible to use the treatment post-launch; as such, they help recover the effect of introducing the new therapy into the real world, sometimes known as the “treatment-on-the-treated” effect.^12^ We limited the pre-launch period to 2 years to mitigate the risk of confounding because of changes over time in treatment or diagnostic protocols. Within both the treated and control groups, we also excluded patients with other metastatic cancers prior to the mCRPC index date.

Using this approach, we identified real-world treated and control patients for each RCT. We then pooled together the samples corresponding to each RCT into a single analytic dataset. Because the RCTs of interest do not necessarily feature unique treatment and/or comparator regimens, patients could appear more than once in the pooled dataset. Below, we explain how we adjusted for this possibility.

### Statistical analysis

For control patients, we defined real-world OS as the time between the index date (defined above) and the date of death. For treated patients, we calculated real-world OS as the time between the date of initial treatment with the novel therapy and date of death. Patients were right-censored if we did not observe a death during their enrollment period in the Optum dataset.

We used Cox proportional hazards to predict real-world OS outcomes as a function of (1) a “treat” indicator equal to 1 if the real-world patient received the treatment arm therapy from a given trial or zero if the patient received the comparator arm therapy; (2) either RCT OS or RCT rPFS hazard ratios as an “offset” term, explained below; and (3) other observed confounders, including patient age at the index date, race (ie, White, Black, or other), year of diagnosis, and patient comorbidity burden measured by the Elixhauser Comorbidity Index in the 6 months prior to first evidence of mCRPC. The fully specified Cox model appears in the Supplementary Methods. We allow each trial to have its own baseline hazard function and clustered standard errors by patient.^13^ Statistical significance is assessed as having a *P* value less than .05.

By including either the RCT OS or RCT rPFS hazard ratios as an “offset” variable in the Cox model,^14^^,^^15^ we can recover the percentage difference in hazard ratios between real-world and RCT settings as $\left(\exp\left(\beta_{1}\right)-1\right)$, where $\beta_{1}$ is the Cox coefficient on the primary independent variable (treat).^10^ A negative adjustment factor predicts that real-world mortality hazard ratios will be less than the corresponding RCT mortality hazard ratios, and vice-versa. Our specification treats the RCT estimates as the reference groups to which real-world outcomes are compared.

We tested the sensitivity of our results to the inclusion of any given trial using the leave-one-out method, where we iteratively dropped 1 trial from the analysis. We then estimated the joint hypothesis that our estimates are not statistically different when excluding any one trial.

Finally, we assessed whether the relationship between real-world OS gains and RCT benefits changed the longer a drug was on the market. We estimated Cox models in 2 ways: (1) comparing pre-launch controls with those treated with the new therapy 0-3 years after launch, and (2) comparing pre-launch controls to those treated 4-6 years after launch. Of note, we do not see new treatment introduction in years 4-6 post launch for any of our drugs, except for sipuleucel-T.

## Results

We documented 5 distinct brand name molecules approved for mCRPC treatment between 2010 and 2019, with 7 corresponding pivotal trials (Table 1). The RCT OS hazard ratio ranged from 0.63 to 0.81 across the trials, with an average of 0.73 (Table 2). Overall, average median survival was 23 months among RCT treatment arm patients and 19 months among RCT comparator arm patients. Across the pivotal trials with RCT rPFS estimates, hazard ratios ranged from 0.17 to 0.66.

**Table 1.** Pivotal trials, approval dates, and inclusion criteria for drugs treating mCRPC.

**Drug name** ^a^	Brand name	Pivotal RCT	Approval date	**Sample restriction applicable to claims data** ^b^
Abiraterone acetate	Zytiga	COU-AA-301	April 28, 2011	Received prior docetaxel
Abiraterone acetate	Zytiga	COU-AA-302	December 10, 2012	No prior chemo
Enzalutamide	Xtandi	PREVAIL	September 10, 2014	No prior chemo
Enzalutamide	Xtandi	AFFIRM	August 31, 2012	Received prior docetaxel
Cabazitaxel	Jevtana	TROPIC	June 17, 2010	Received prior docetaxel
Sipuleucel-T	Provenge	IMPACT	April 29, 2010	None
Radium-223	Xofigo	ALSYMPCA	May 15, 2013	None

Data on which drugs are approved for mCRPC come from the National Cancer Institute and National Comprehensive Cancer Network Treatment Guidelines. Pivotal RCT, approval dates, and additional sample restrictions are from the Food and Drug Administration drug labels and published studies for each RCT.

a Because we follow new molecules, we do not include Yonsa (abiraterone acetate), which cited the same pivotal RCT trials in FDA approval as the pivotal trials used to evaluate Zytiga.

b We noted only those sample restrictions that can be identified in claims data, there are many additional restrictions relevant to these clinical trials.

**Table 2.** Overall survival (OS) and radiographic progression-free survival in pivotal trials.

Pivotal trial	**Treatment group** ^a^	**Control group** ^a^	**Median OS** **treat**	Median OS control	**OS** **HR**	Median rPFS treat	Median rPFS control	**rPFS** **HR**
COU-AA-301	Abiraterone acetate + prednisone	Placebo + prednisone	15.8	11.2	0.74 (0.64, 0.86)	5.6	3.6	0.66 (0.58, 0.76)
COU-AA-302	Abiraterone acetate + prednisone	Placebo + prednisone	34.7	30.3	0.81 (0.70, 0.93)	NR	8.28	0.43 (0.35, 0.52)
PREVAIL	Enzalutamide + ADT	Placebo + ADT	35.3	31.3	0.77 (0.67, 0.88)	NR	3.7	0.17 (0.14, 0.21)
AFFIRM	Enzalutamide + ADT	Placebo + ADT	18.4	13.6	0.63 (0.53, 0.75)	8.3	2.9	0.40 (0.35, 0.47)
TROPIC	Cabazitaxel+ prednisone	Mitoxantrone+ prednisone	15.1	12.7	0.70 (0.59, 0.83)			
IMPACT	Sipuleucel-T	Placebo	25.8	21.7	0.78 (0.62, 0.98)			
ALSYMPCA	Radium-223 + SOC	Placebo + SOC	14.9	11.3	0.70 (0.56, 0.88)			

Data from FDA drug approval labels and the published studies on each RCT.

a ADT refers to androgen deprivation therapy. SOC refers to the best standard of care.

In the Optum data, we identified 4850 patients with mCRPC. Table 3 shows sample counts after we applied the additional sample restrictions noted in Table 1 and identified real-world use of the treatment and comparator regimens listed in Table 2. Because the mCRPC treatments of interest were approved between 2010 and 2014, and the Optum data begin in 2009, real-world control group samples were larger when drawn from the period after a new therapy launched than from the period before launch. There were no real-world patients using the regimen in the comparator arm for the cabazitaxel RCT (ie, mitoxantrone), so we dropped the trial (TROPIC) in subsequent real-world OS estimates.

**Table 3.** Real-world sample counts in treatment and comparator regimens in pivotal RCTs.

Drug name	Pivotal RCT	**Patients in** **treatment**	**Patients in** **post-launch control**	**Patients in** **pre-launch control**
Abiraterone acetate	COU-AA-301	666	2599	91
Abiraterone acetate	COU-AA-302	1196	700	79
Enzalutamide	PREVAIL	1367	1713	129
Enzalutamide	AFFIRM	122	572	56
Sipuleucel-T	IMPACT	141	4108	41
Radium-223	ALSYMPCA	326	4121	157

Data from Optum from 2009 to 2020. Control groups consist of patients receiving the comparator regimen in all years after the new treatment launch (post-launch), or 2-years prior to new treatment launch (pre-launch), respectively. For trials on chemotherapy naïve patients, we focused on patients who had not taken carboplatin, cabazitaxel, cisplatin, docetaxel, etoposide, or mitoxantrone prior to the index mCRPC dates.

Age distributions were similar for real-world patients and those in the corresponding RCTs. However, real-world samples featured higher shares of Blacks and Asians (Table S3). The months of follow-up in the real-world data generally exceeded that in RCTs, except in the cases of the COU-AA-302, IMPACT, and ALSYMPCA trials (Table S4).

Table 4 specifies the real-world OS hazard ratios across all RCT treatment and comparator regimens. When using post-launch controls (Columns (1) and (2)), real-world OS ranged from 0.67 to 1.32. However, when comparing treated patients to pre-launch controls, real-world OS ranged from 0.34 to 1.06. In nearly all cases, real-world OS hazard ratios were lower than the RCT OS hazard ratios, suggesting that the magnitude of survival gains associated with treatment in the real-world exceeded RCT treatment effects. Real-world OS hazard ratios using pre-launch controls were also considerably closer to RCT OS hazard ratios (Table S5). Adjusting for age, race, year of diagnosis, and patient comorbidities did not significantly change real-world OS hazard ratios.

**Table 4.** Real-world overall survival estimates corresponding to treatment and comparator regimens in pivotal RCTs.

Drug name	Pivotal RCT	Real-world OS using Post-launch controls		Real-world OS using Pre-launch controls	
		Unadjusted	Adjusted	Unadjusted	Adjusted
		(1)	(2)	(3)	(4)
Abiraterone acetate	COU-AA-301	1.26*	1.25*	0.77*	0.83
		[1.13, 1.39]	[1.11, 1.40]	[0.61, 0.98]	[0.57, 1.21]
Abiraterone acetate	COU-AA-302	1.32*	1.38*	1.04	0.81
		[1.15, 1.50]	[1.20, 1.58]	[0.80, 1.36]	[0.56, 1.16]
Enzalutamide	PREVAIL	0.88*	0.88*	0.88	0.63*
		[0.80, 0.99]	[0.79, 0.97]	[0.71, 1.09]	[0.47, 0.85]
Enzalutamide	AFFIRM	1.19	1.16	0.75	0.65
		[0.94, 1.51]	[0.89, 1.50]	[0.52, 1.07]	[0.30, 1.40]
Sipuleucel-T	IMPACT	0.69*	0.67*	0.34*	0.72
		[0.56, 0.84]	[0.54, 0.83]	[0.23, 0.51]	[0.35, 1.51]
Radium-223	ALSYMPCA	1.30*	1.27*	1.06	0.96
		[1.14, 1.48]	[1.11, 1.44]	[0.85, 1.31]	[0.60, 1.55]

Data from Optum from 2009 to 2020. Real-world overall survival (OS) hazard ratios (HRs) estimates from Cox proportional hazards models. Control groups consist of patients receiving the comparator regimen in all years after the new treatment launch (post-launch), or 2 years prior to new treatment launch (pre-launch), respectively. Columns (2) and (4) adjusts for age, race, year of diagnosis, and Elixhauser Comorbidity Index.

* Indicates that the hazard ratio is statistically different from 1 with *P* < .05.

**Figure 1.**
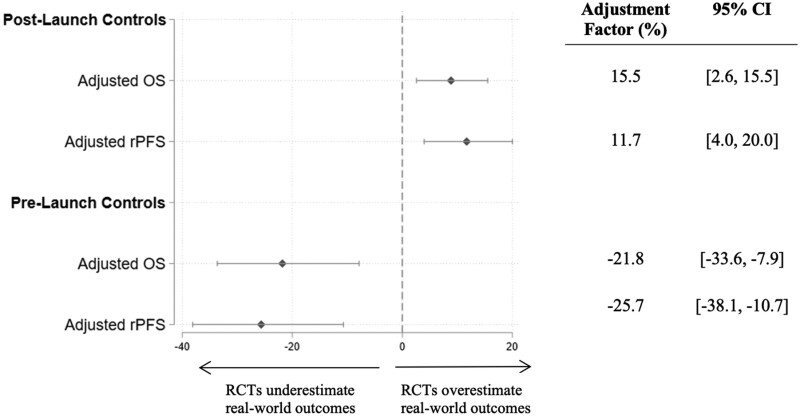
Relationship between real-world OS and RCT OS and RCT rPFS in mCRPC. Each dot shows the adjustment factor and corresponding 95% confidence interval from separate Cox proportional hazards models. The Adjusted OS models offset using RCT OS, whereas the Adjusted rPFS models offset using RCT rPFS. The Post-Launch and Pre-Launch compare treated patients with controls patients identified in years after, or 2 years before, the new therapies’ launch dates, respectively. All models controlled for year of diagnosis, patient age at index date, prediagnosis Elixhauser comorbidity scores, and patient race. We stratified by trial and cluster standard errors by patient.

We show the relationship between real-world OS and RCT OS hazard ratios, and that between real-world OS and RCT rPFS hazard ratios in Figure 1. The full regression results are displayed in Table S6. When using the post-launch controls, real-world OS treatment associations were 16% less than RCT OS benefits (95% CI = 3% to 16%), and real-world OS treatment associations were 12% less than RCT rPFS benefits (95% CI = 4% to 20%). However, when using pre-launch controls to mitigate confounding from nonrandom treatment selection, real-world OS treatment associations were 22% (95% CI = 8% to 36%) higher than RCT OS benefits and 26% (95% CI = 11% to 38%) higher than RCT rPFS benefits, meaning that the treatments in the real-world setting were associated with larger effectiveness than in the RCT setting.

**Figure 2.**
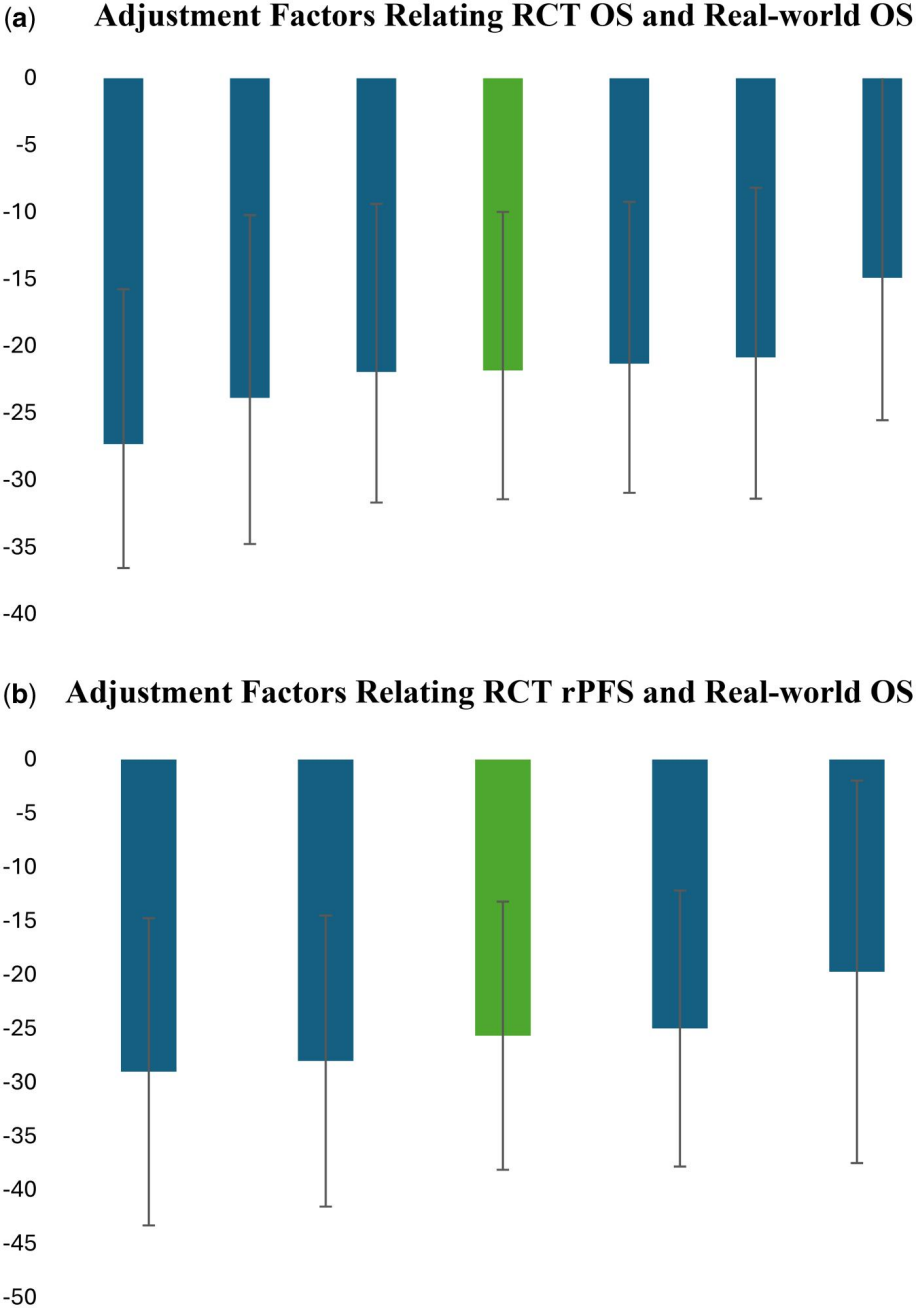
Sensitivity of estimates to individual trial estimates. The darker bars show the adjustment factor estimate and corresponding 95% confidence interval form “all but one” clinical trial. The lighter bar shows the adjustment factor and corresponding 95% confidence interval using all trials in Table 2. All models controlled for year of diagnosis, patient age at index date, prediagnosis Elixhauser comorbidity scores, and patient race. We stratified by trial and cluster standard errors by patient.

These estimates were not sensitive to the inclusion or exclusion of any specific trial. As shown in Figure 2, the adjustment factors from the leave-on-out method were similar to the baseline estimates. The joint-hypothesis test yielded significance levels of 21% and 67%, indicating that there is no statistical difference in the estimates from the inclusion of any one given trial.

**Figure 3.**
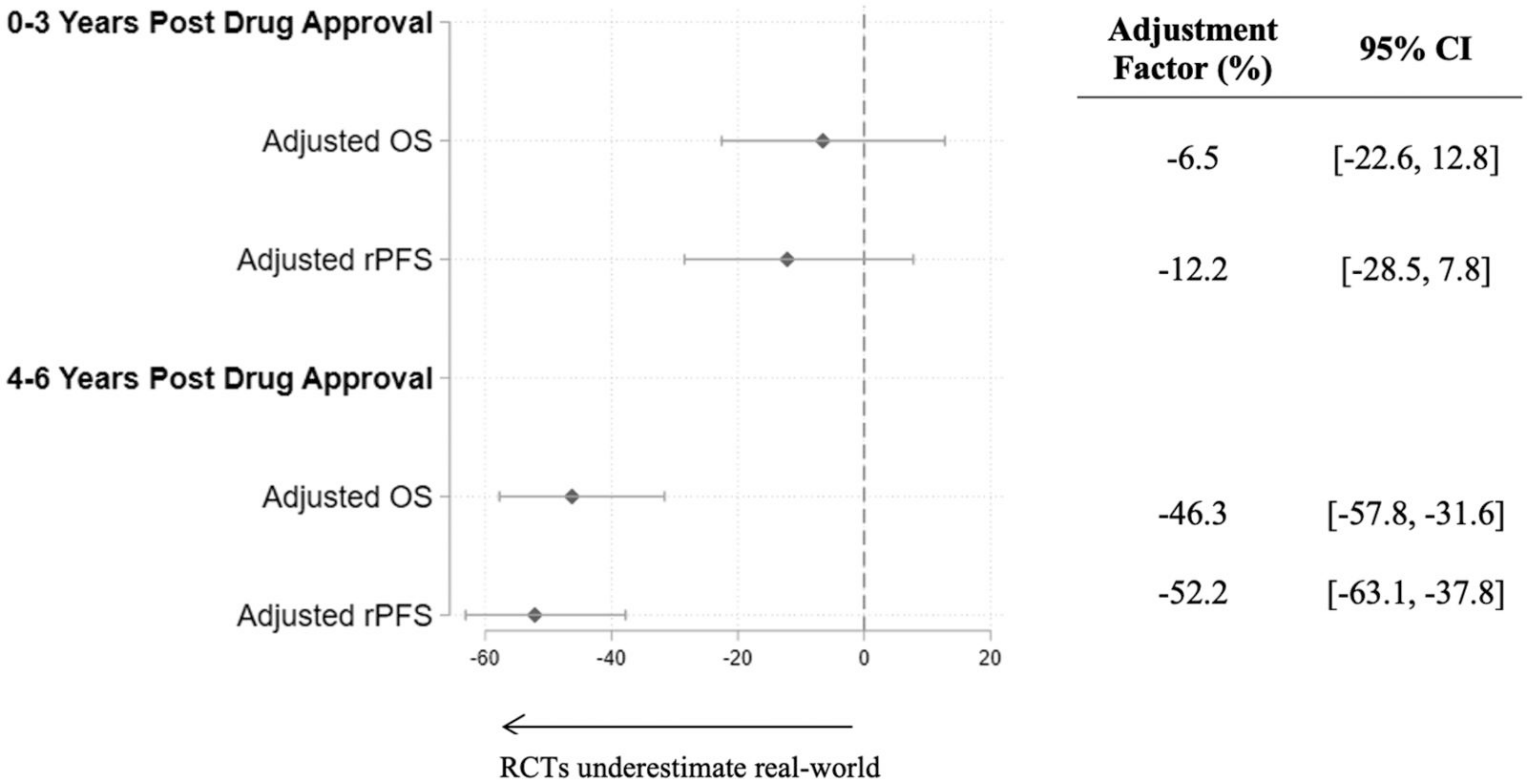
Relationship between real-world OS and RCT OS and RCT rPFS in mCRPC over time. Each dot shows the adjustment factor and corresponding 95% confidence interval from separate Cox proportional hazards models. The Adjusted OS models offset using RCT OS, whereas the Adjusted rPFS models offset using RCT rPFS. The control group in all models consists of patients receiving the comparator regimens in the 2 years prior to the corresponding treatments’ launch dates. The 0-3 Years, 4-6 Years, examines patients who were treated with the treatment of interest 0-3 years or 4-6 years after the drug had launched. All models controlled for year of diagnosis, patient age at index date, prediagnosis Elixhauser comorbidity scores, and patient race. We stratified by trial and cluster standard errors by patient.

Using our long post-treatment period, we depict in Figure 3 how the relationship between real-world OS and RCT OS or RCT rPFS changed over time. The effectiveness associated with real-world treatment (estimated by comparing treatment to pre-launch controls) grew stronger the longer a drug was on the market. Among patients receiving real-world treatment 0-3 years after a new therapy launched, real-world OS gains associated with treatment were 7% and 13% higher than RCT OS and RCT rPFS, respectively. These real-world OS gains associated with treatment grew to 46% and 52% higher than RCT OS and RCT rPFS, among real-world patients treated 4-6 years after a new therapy had launched.

## Discussion

We consistently found that real-world hazard ratios were larger when using post-launch controls relative to pre-launch controls. This finding comports with the possibility of nonrandom selection into therapy of the sickest patients with the greatest need for therapy; if true, this dynamic would bias real-world effectiveness estimates toward zero. The pattern of results also underscores the importance of accounting for differences between patients who do and do not receive a new therapy in the real-world setting.

When we rely on pre-launch controls, we find that the therapies of interest exhibited real-world treatment associations stronger than their corresponding RCT estimates. There are several possible explanations of this finding. Real-world patients on treatment may be healthier than RCT participants in ways that cannot be measured in our data. On the other hand, RCT participants on comparator therapies may be healthier than real-world patients. Alternatively, there may be differences in treatment management across the RCT and real-world settings; for example, the real-world data may reflect the use of newer and better disease-management protocols than were available during the respective trials. Further research is needed to ascertain the relevant mechanisms.

Existing meta-analyses on the relationship between RCT efficacy and real-world effectiveness have yielded mixed results, with some indicating lower real-world effectiveness, others finding no efficacy-effectiveness gap, and still others showing higher incremental gains in real-world settings compared with RCT settings.^6-10^ The mixed results may be driven by differences across the studied patient populations, selection bias in therapy choice, and/or variation across cancer indications and therapies. Additionally, our findings differ somewhat from Lakdawalla et al. (2017), who found that real-world OS was similar to RCT OS across 5 (nonprostate) tumor types.^10^ However, they did find better real-world performance for colorectal cancer and for patients using the treatments they studied as second- or third-line therapies.^10^

Although it is unclear why these therapies performed on average better in the real world than in RCT settings, we found that the longer the drug was on the market, the larger the real-world survival gains became. It is possible that physicians improved their identification of patients who would benefit the most from taking the new therapy or that earlier diagnosis led to healthier patients taking up the drug over time. Alternatively, there may have been slower take-up shortly after the new drug launched, suggesting that patients who received the new therapy earlier in the drug’s marketing date were different from those receiving the drug at later time periods. Over time, physicians may become more adept at managing side-effects and thus keeping patients on treatment at potentially higher doses, thereby maximizing benefits. Finally, it is possible that later-treated patients had access to subsequent treatments that additionally improved survival.

Our results highlight the importance of closely studying differences in baseline health status between trial participants and real-world mCRPC patients, and how these differences evolve as physicians gain more experience with a new therapy. Additionally, our data suggest the need for careful consideration by providers and insurers attempting to infer real-world benefit from RCT data of the therapies we studied.

Our study has several limitations. We relied on claims data to analyze real-world survival outcomes. Optum is limited to a subset of patients with commercial or Medicare Advantage insurance provided by 1 insurer. As such, our results may not generalize to other populations, such as the uninsured or those with Medicaid or traditional Medicare insurance. Additionally, very few patients in the data had laboratory test results, which made it difficult to rely on prostate-specific antigen (PSA) tests to identify mCRPC patients. Instead, we used predominantly non-PSA-related criteria, which required accurate diagnoses, procedure, and drug code information in the claims data.^16^ We did not have access to real-world rPFS data. We also cannot eliminate bias from real-world treatment decisions or from changing care patterns, and we cannot identify the relationship between real-world and RCT outcomes for patients not treated in the real world. Given that our data did not include detailed information on socioeconomic status, future research using alternate data sources that provide both mortality and detailed socioeconomic data are needed. Finally, we are unable to capture newer approved treatments, such as poly-ADP ribose polymerase inhibitors and other recent innovations in the standard of care.

In light of constrained healthcare budgets and the search for treatment strategies that optimize value for patients, rigorous comparisons of real-world and clinical trial outcomes have become increasingly important. We found that real-world OS gains associated with a set of mCRPC treatments were consistently higher than those observed in corresponding RCTs. In other words, both RCT OS and RCT rPFS estimates lie below real-world associations between treatment and survival gain. We also found suggestive evidence that real-world effectiveness associated with treatment grew over time. These data leave open the possibility that mCRPC therapies are performing at least as well in the real world as in their pivotal RCTs.

## Supplementary Material

pkaf018_Supplementary_Data

## Data Availability

Data for this article comes from a combination of publicly available and restricted use data sources. The publicly available data sources include the National Cancer Institute, National Comprehensive Cancer Network Treatment Guidelines, and Federal Trade Commission. The data collected from those sources are shared in Tables 1 and 2. The restricted use data come from the Optum Clinformatics Extended DataMart Databases, and our data use agreement prohibits sharing this data. However, the code used to analyze this data is available on Github, and information on how to access the Optum data can be found here: https://www.optum.com/business/life-sciences/real-world-data/claims-data.html.
