# Supplementary material for: Do trial benefits predict real-world gains in metastatic castration resistant prostate cancer
Source: JNCI Cancer Spectr. 2025 Feb 13;9(2):pkaf018. doi: 10.1093/jncics/pkaf018 (PMC11879356; doi:10.1093/jncics/pkaf018)
Supplement: pkaf018_Supplementary_Data [file pkaf018_supplementary_data.docx]

**Supplementary Materials**

**Supplementary Results**

**Supplementary Table 1. Sample Selection Criteria for Patients with mCRPC**

| **Step 1: IDENTIFY PATIENTS WITH METASTATIC PROSTATE CANCER (mPC)** | |
| --- | --- |
| Male Patients with 2 or more outpatient or 1 or more inpatient diagnoses of Prostate Cancer | 492,668 |
| 1 or more diagnoses for metastatic disease on or after first observed prostate cancer diagnosis | 63,579 (12.9%) |
| ***No claims for metastatic disease prior to first observed prostate cancer diagnosis*** | 58,179 (91.5%) |
| **Step 2: IDENTIFY EVIDENCE OF CASTRATION RESISTANCE IN CLAIMS** | |
| EVIDENCE FROM MEDICAL CLAIMS |  |
| Any diagnosis for hormone resistance | 2,598 |
| Any diagnosis for rising PSA after surgical castration | 50 |
| Any diagnosis for rising PSA during an episode of medical castration | 541 |
| Initial metastatic diagnosis made 90 or more days after surgical castration | 103 |
| Initial metastatic diagnosis made 90 or more days after medical castration | 1,621 |
| EVIDENCE FROM MEDICATION CLAIMS |  |
| Any claim for cabazitaxel, mitoxantrone, radium-223, or sipuleucel-T | 4,389 |
| Any claim for abiraterone acetate before June 2017 or initial claim for abiraterone acetate ≥90 days after initiation of ADT | 6,193 |
| Any claim for enzalutamide before December 2019 or initial claim for enzalutamide ≥90 days after initiation of ADT | 6,314 |
| Earliest claim for docetaxel ≥90 days after initiation of ADT | 5,200 |
| ***Total mPC patients with evidence of castration resistance*** | ***15,256 (26.2%)*** |
| **Step 3. ADDITIONAL DIAGNOSIS-BASED EXCLUSIONS** | |
| No claims for hormone sensitive malignancy status on or after first evidence of castration resistance | 14,564 (95.4%) |
| No claims for other cancer prior to or on the index date* | 7,303 (50.1%) |
| **Step 4. REMOVE PATIENTS WITH LIMITED CONTINUOUS ENROLLMENT PERIOD** | |
| ***180 days of continuous enrollment prior to and 180 days of continuous enrollment (or death) following the index date.*** | ***4,850 (66.4%)*** |

**Notes:** Numbers are counts of unique patients; values in parentheses are shares of the prior data step.

*Index date is defined as the later of the first date of diagnosis of metastatic disease or the first date with evidence of castration resistance.

^Specific definitions used to construct the sample are available in Appendix Table A2.

**Supplementary Table 2. Definitions for Inclusion and Exclusion Criteria in Construction of**

**mCRPC sample**

| **Inclusion or Exclusion Criteria** | **Definition in CDM dataset** |
| --- | --- |
| Prostate Cancer | ICD-9 code = 185*  ICD-10 code = C61* |
| Metastatic Disease | ICD-9 code = 196*, 199.1*, 209.7*  ICD-10 code = C77*, C78*, C79*, C80.0, C7B* |
| Other Cancers | ICD-9 code = 14*, 15*, 160*-165*, 170*-189*, 200*-208*, 209.0*, 209.1*, 209.2*, 209.3*, 190-193*, 195*, 199*, 258.0*, 789.51  ICD-10 code = C01*-C26*, R180*, C30*-C58*, C60*, C62*-C79*, C80*-C96*, C7A*, C7B*, E31.20, E31.21, E31.22, E31.23 |
| Hormone Resistance Diagnosis | ICD-9 code = n/a  ICD-10 code = Z19.2 |
| Rising PSA Diagnosis | ICD-9 code = n/a  ICD-10 code = R97.21 |
| Surgical Castration | Evidence of 2 unilateral [Procedure codes: 0VT9, 0VTB, 623, 54520 and modifier 50, 54522, 54530]  or  1 bilateral orchiectomy [Procedure codes: 0VTC, 624, 54521, 54690] |
| Medical Castration | 90 days of leuprolide, triptorelin, goserelin, histrelin, degarelix, relugolix with no more than 30 days without those drugs |
| Diagnosis of hormone sensitive malignancy status | ICD-9 code = n/a  ICD-10 code = Z19.1 |
| ADT | Fills or injections of leuprolide, triptorelin, goserelin, histrelin, degarelix, relugolix |
| Drugs solely recommended for mCRPC | Fills or injections of Enzalutamide,  Diethylstilbestrol Diphosphate, Estramustine Phosphate, Polyestrodiol Phosphate, Abiraterone Acetate, Cabazitaxel, Mitoxantrone, Cisplatin, Carboplatin, Etoposide, Pembrolizumab, Sipuleucel-T, Radium-223 |

**Supplementary Table 3. Comparing RCT and Real-World Demographics**

| **Drug Name** | **Pivotal**  **RCT** | **RCT Age Median (Range)** | **RCT**  **Race** | **RW Age  Median (Range)** | **RW**  **Race** |
| --- | --- | --- | --- | --- | --- |
| Abiraterone Acetate | COU-AA-301 | 69 (39-95) | 93% White  3.6% Black  1.7% Asian  1.6% Other | 72 (46-90) | 75% White  20% Black  1.9% Asian  2.7% Other |
| Abiraterone Acetate | COU-AA-302 | 70* | 95% White  2.8% Black  0.7% Asian  1.1% Other | 73 (47-90) | 74.2% White  21.1% Black  2.2% Asian  2.5% Other |
| Enzalutamide | PREVAIL | 71 (42-93) | 77% White 10% Asian 2% Black  11% Other | 74 (48-90) | 69.1% White  25.3% Black  3.1% Asian  2.4% Other |
| Enzalutamide | AFFIRM | 69 (41-92) | 92.7% White  3.9% Black  1.1% Asian  2.1% Other | 69 (46-87) | 66.9% White  27.3% Black  3.8% Asian  2.3% Other |
| Sipuleucel-T | IMPACT | 72 (49-91) | 89.4% White  6.7% Black  3.8% Other | 70.5 (52-88) | 83% White  15.6% Black  1.6% Other |
| Radium-223 | ALSYMPCA | 71 (44-94) | 94% White  2% Black  4% Asian  <1% Other | 72 (46-90) | 80% White  15.5% Black  1.9% Asian  2.4% Other |

*Notes:* Randomized controlled trial (RCT) data taken from the most recent FDA drug labels and supplemented with the published study as needed. *We note that there were no published age range for COU-AA-302. Real-world (RW) data is from Optum Clinformatics, which censors age at 90 for privacy reasons.

**Supplementary Table 4: Additional Summary Statistics From RCT and RW Datasets**

| **Drug Name** | **Pivotal RCT** | **RCT**  **Total**  **Obs**  **(1)** | **RCT Median**  **Follow-Up**  **(months)**  **(2)** | **RW**  **Total**  **Obs**  **(3)** | **RW Missing Data on Race**  **(4)** | **RW Censoring**  **(months)**  **(5)** |
| --- | --- | --- | --- | --- | --- | --- |
| Abiraterone Acetate | COU-AA-301 | 1195 | 20.2 | 757 | 204 | 23 |
| Abiraterone Acetate | COU-AA-302 | 1088 | 49.2 | 1275 | 197 | 25 |
| Enzalutamide | PREVAIL | 1717 | 20.0 | 1496 | 182 | 22 |
| Enzalutamide | AFFIRM | 1199 | 14.4 | 178 | 45 | 27 |
| Sipuleucel-T | IMPACT | 512 | 34.1 | 182 | 60 | 30 |
| Radium-223 | ALSYMPCA | 921 | 36.0 | 483 | 115 | 23 |

*Notes:* Randomized controlled trial (RCT) taken from the most recent FDA drug labels and supplemented with the published study as needed. Real-world (RW) data is from Optum Clinformatics.

**Supplementary Table 5: RCT and RW Overall Survival Hazard Ratios**

| Drug Name | Pivotal RCT | RCT OS Hazard Ratios |  | Real-World OS Using  Post-Launch Controls | |  | Real-World OS Using  Pre-Launch Controls | |
| --- | --- | --- | --- | --- | --- | --- | --- | --- |
|  |  |  |  | **Unadjusted** | **Adjusted** |  | **Unadjusted** | **Adjusted** |
|  |  | **(1)** |  | **(2)** | **(3)** |  | **(4)** | (5) |
| Abiraterone Acetate | COU-AA-301 | 0.74 |  | 1.26 | 1.25 |  | 0.77 | 0.83 |
|  |  | [0.64, 0.86] |  | [1.13, 1.39] | [1.11, 1.40] |  | [0.61, 0.98] | [0.57, 1.21] |
| Abiraterone Acetate | COU-AA-302 | 0.81 |  | 1.32 | 1.38 |  | 1.04 | 0.81 |
|  |  | [0.70, 0.93] |  | [1.15, 1.50] | [1.20, 1.58] |  | [0.80, 1.36] | [0.56, 1.16] |
| Enzalutamide | PREVAIL | 0.77 |  | 0.88 | 0.88 |  | 0.88 | 0.63 |
|  |  | [0.67, 0.88] |  | [0.80, 0.99] | [0.79, 0.97] |  | [0.71, 1.09] | [0.47, 0.85] |
| Enzalutamide | AFFIRM | 0.63 |  | 1.19 | 1.16 |  | 0.75 | 0.65 |
|  |  | [0.53, 0.75] |  | [0.94, 1.51] | [0.89, 1.50] |  | [0.52, 1.07] | [0.30, 1.40] |
| Sipuleucel-T | IMPACT | 0.78 |  | 0.69 | 0.67 |  | 0.34 | 0.72 |
|  |  | [0.62, 0.98] |  | [0.56, 0.84] | [0.54, 0.83] |  | [0.23, 0.51] | [0.35, 1.51] |
| Radium-223 | ALSYMPCA | 0.70 |  | 1.30 | 1.27 |  | 1.06 | 0.96 |
|  |  | [0.56, 0.88] |  | [1.14, 1.48] | [1.11, 1.44] |  | [0.85, 1.31] | [0.60, 1.55] |

*Notes:* Randomized controlled trial (RCT) taken from the most recent FDA drug labels and supplemented with the published study as needed. Real-world (RW) data is from Optum Clinformatics. Adjusted HRs control for age, race, year of diagnosis, and Elixhauser Comorbidity Index.

**Supplementary Table 6: Additional Summary Statistics From RCT and RW Datasets**

|  | **Post-Launch Controls** | | **Pre-Launch Controls** | |
| --- | --- | --- | --- | --- |
|  | (1) | (2) | (3) | (4) |
| Adjusted OS | 0.085 |  | -0.246 |  |
|  | [0.025, 0.144] |  | [-0.410, -0.082] |  |
| Adjusted rPFS |  | 0.111 |  | -0.297 |
|  |  | [0.039, 0.182] |  | [-0.480, -0.113] |
| Year of Diagnosis | -0.03 | -0.018 | 0.011 | 0.019 |
|  | [-0.054, -0.007] | [-0.045, 0.010] | [-0.020, 0.042] | [-0.014, 0.052] |
| Age | 0.034 | 0.033 | 0.031 | 0.032 |
|  | [0.029, 0.040] | [0.028, 0.040] | [0.025, 0.038] | [0.025, 0.039] |
| Elixhauser | 0.025 | 0.026 | 0.002 | 0.006 |
|  | [0.015, 0.035] | [0.015, 0.036] | [-0.011, 0.015] | [-0.007, 0.019] |
| 1(Black) | -0.178 | -0.154 | -0.394 | -0.310 |
|  | [-0.2318, -0.038] | [-0.306, -0.001] | [-0.575, -0.212] | [-0.493, -0.126] |
| 1(White) | -0.086 | -0.056 | -0.372 | -0.295 |
|  | [-0.200, 0.028] | [-0.181, 0.068] | [-0.508, -0.236] | [-0.436, -0.154] |
| OBS | 17,610 | 8929 | 4371 | 3706 |

*Notes:* Each column shows estimates from a separate Cox proportional hazard model. The “Adjusted OS” models offset using RCT OS, while the “Adjusted rPFS” models offset using RCT rPFS. The “Post-Launch,” and “Pre-Launch,” compare treated patients to controls patients identified in years after, or 2-years before the new therapies’ launch dates, respectively. All models controlled for year of diagnosis, patient age at index date, and patient race, and patient Elixhauser Comorbidity Index. We stratified by trial and cluster standard errors by patient.

**Supplementary Methods**

To measure the relationship between real-world treatment effectiveness in Optum as measured by OS and predicted treatment efficacies from RCTs based on either OS or rPFS, we estimated Cox proportional hazard models. Specifically, for patient $i$in Optum and drugs therapies used in RCT trial $j$*,* we specified:

$\lambda_{OS,i,j}^{RW}\left( t \right)=\lambda_{o,i,j}\left( t \right){\exp\left( \beta_{1}+\ln\left[ HR_{RCT,j} \right] \right)}^{T_{ij}}\exp\left( X_{i} \right)$ (1)

The terms were defined as follows:

- $\lambda_{OS,i,j}^{RW}\left( t \right)$ = real-world OS hazard function for patient $i$using the therapies (treatment or comparator) in trial j
- $\lambda_{0,i,j}$= associated baseline Cox hazard function
- $HR_{RCT,j}$= RCT hazard ratio (either OS or rPFS)
- $T_{ij}$ = indicator equal to one if patient $i$ in Optum received the treatment arm therapy from trial $j$or zero if patient $i$ in Optum received the comparator arm therapy from trial
- $X_{i}$= vector of patient-level characteristics including year of diagnosis, age at the index date, and race indicators

Equation (1) makes clear that the log transformed RCT hazard ratios acts as an offset term. Because we estimate Equation (1) over several trials where patients can serve as a control group in more than one trial, we stratified by the RCT based on the treatment patients in Optum received, and we clustered standard errors by patient.

We estimated Equation (1) twice: once with $HR_{RCT,j}$ as the RCT OS hazard ratios, and again with $HR_{RCT,j}$ as the RCT rPFS hazard ratios. The regression coefficient $\beta_{1}$ can be used to calculate the percentage difference between real-world mortality hazard ratios and clinical trial hazard ratios, given by $\exp\left( \beta_{1} \right)-1$.
